# Effect of Intraoperative Phenylephrine Infusion on Redistribution Hypothermia During Cesarean Delivery Under Spinal Anesthesia

**DOI:** 10.16966/2470-9956.103

**Published:** 2015-12-19

**Authors:** EJ Hilton, SH Wilson, BJ Wolf, W Hand, L Roberts, L Hebbar

**Affiliations:** 1 Department of Anesthesia and Perioperative Medicine, Medical University of South Carolina, USA; 2 Department on Public Health Sciences, Medical University of South Carolina, USA

**Keywords:** Phenylephrine, Hypothermia, Neuraxial anesthesia

## Abstract

An observational clinical study to evaluate the effect of phenylephrine infusion on maternal temperatures during scheduled cesarean delivery under spinal anaesthesia was conducted in 40 ASA physical status II parturients. Following placement of spinal anesthesia, phenylephrine infusion was initiated at 40 μg/min and titrated to maintain mean arterial pressure within 20 percent of baseline. Maternal oral temperature, heart rate, and blood pressure were measured at baseline, spinal placement, every 10 minutes thereafter for 60 minutes. Phenylephrine dose received was documented every ten minutes. The range in maternal temperature change was 0.06–0.29°C. The lowest recorded temperature was 36.3°C. Decreased maternal temperature was associated with duration of anesthesia and cumulative phenylephrine dose in a univariate model (P<0.001 for all). The multivariable model showed an association between a greater decrease in maternal temperature with larger doses of phenylephrine being administered.

## Introduction

Heat is stored in the body in two compartments: core and peripheral. Normal core temperature averages 37°C with peripheral temperatures measuring 2°C cooler. Thermoregulatory mechanisms with an interthreshold range of 0.2–0.4°C maintain temperature in awake patients [1]. During the perioperative period, the inability to maintain body temperature is attributed to cold ambient temperature and disruption of thermoregulatory responses [2,3]. Anesthetic agents affect perioperative hypothermia by lowering the hypothalamic thermoregulatory set point for shivering and by increasing core-to-peripheral heat redistribution through vasodilation [2,3]. This redistribution component occurs to a large extent during the first hour following induction of anesthesia and is a major cause of hypothermia in short surgical procedures [4].

In the perioperative setting, hypothermia has been identified as an independent risk factor for adverse outcomes. It has been linked to elevated transfusion requirements due to coagulopathy, impaired wound healing, higher infection rates, prolonged postoperative recovery, adverse cardiac events with increased oxygen consumption, increased postoperative nausea and vomiting, shivering, and worse patient comfort scores [2,5,6]. Despite these known morbidities [5], perioperative hypothermia continues to be a problem with an estimated incidence of 20–30% for general surgical procedures [7–9]. Moreover, hypothermia for Cesarean delivery under spinal anesthesia has a reported incidence greater than 77% for temperatures less than 36°C and 60% for temperatures less than 35.5°C [10,11].

In the United States, Cesarean delivery is the most common indication for laparotomy with a rate greater than 31.3% [10–12]. For these procedures, neuraxial anesthesia is preferred to general anesthesia for the benefits of maternal awareness, avoidance of maternal airway instrumentation, and decreased incidence of neonatal depression. However, similar to general anesthesia, neuraxial techniques are also associated with hypothermia [10,11], and prevention strategies have been evaluated. One perioperative strategy utilization of phenylephrine, a selective α_1_-adrenergic receptor agonist, did demonstrate a reduction in intraoperative hypothermia in elective orthopedic cases under spinal anesthesia [13]. To our knowledge, this intervention for hypothermia prevention has not been examined in the obstetric surgical population.

Our hypothesis was that a continuous infusion of phenylephrine would decrease the degree of intraoperative hypothermia during Cesarean delivery under subarachnoid anesthesia. The primary outcome of this study was the change in maternal temperature and secondary endpoints included the incidence of shivering, nausea and emesis, blood loss, and surgical site infection.

## Methods

Ethical approval for this study was provided by the Institutional Review Board for Human Research and Office of Research Integrity of the Medical University of South Carolina, Charleston, SC (Vice-Chairman Steven Swift) on 3 January 2012.

Following IRB approval, forty parturients underwent written informed consent and were enrolled in this observational, prospective study with patient enrollment from April 2012 to June 2013. Inclusion criteria included non-laboring parturients 18 to 40 years old scheduled for Cesarean delivery with spinal anesthesia. Exclusion criteria included ASA physical status score of III or greater, thyroid disease, Raynaud’s syndrome, autonomic neuropathy, hypertensive disease of pregnancy, preeclampsia, BMI greater than 35, and non-English speaking.

One liter of lactated Ringers solution was co-loaded, over 15 minutes, via free drip using 10 gtt/mL IV tubing with open roller clamp, through a fluid warmer set at 40°C during spinal anesthesia placement (hyperbaric bupivacaine 12 mg, fentanyl 15 μg, and morphine 0.2 mg) in an operating room maintained between 25–26°C. Following subarachnoid injection, a phenylephrine infusion was initiated at 40 μg per minute and titrated to maintain mean arterial pressure (MAP) within 20% of baseline throughout the case. Patients were positioned supine with left uterine displacement. Loss of discrimination to cold temperature was used to determine level of anesthetic block and additional warm blankets housed in a warmer set at 50°C were provided based on patients’ comfort. Data on maternal oral temperature was noted by sublingual measurement using a Welch-Allyn SureTempPlus 692 digital temperature probe. Heart rate and blood pressure were collected at baseline and every ten minutes following spinal placement for sixty minutes. Phenylephrine dose received was documented every ten minutes. The incidence of maternal hypotension, bradycardia, patient’s endorsement of nausea, witnessed episodes of vomiting, shivering, blood loss (visually estimated by the performing anesthesiologist), and wound infection were also recorded to analyze as secondary outcomes. Hypotension and bradycardia were defined as a decrease of 20% from baseline. Nausea and vomiting was documented either as absent or present. Shivering was documented using the Bedside Shivering Assessment Scale (BSAS) from 1 to 4: (1) None: no shivering noted on palpation of the masseter, neck or chest wall; (2) Mild: shivering localized to the neck and/or thorax only; (3) Moderate: shivering involves gross movement of the upper extremities (in addition to neck and thorax); (4) Severe: shivering involves gross movements of the trunk and upper and lower extremities [14]. Chart review at two weeks was performed to determine the incidence of wound infection.

A sample size of 40 patients was calculated to detect a correlation of 0.43 with 80% power (α=0.05). Additionally, our multivariate model that included 4 covariates from 40 patients is consistent with Harrell’s rule of thumb that at least 10 observations per predictor are needed to prevent over-fitting for multivariable modeling [15,16] of a continuous outcome (i.e. maternal temperature). Study power is likely greater given repeated measures were taken on each patient.

Change in maternal temperature, maternal heart rate, and maternal mean arterial pressure were analyzed using linear mixed regression models accounting for repeated measures on each subject. First order auto-regressive, heterogeneous auto-regressive, compound symmetry and unstructured covariance structures were considered and the best structure selected by comparing Akaike information criterion (AIC) and Bayesian information criterion (BIC) for each model. Secondary outcomes included estimated total blood loss, occurrence of shivering during the procedure, and occurrence of nausea or vomiting during the procedure. The association between cumulative phenylephrine dose before PACU and blood loss was examined using Pearson’s correlation. The Mann-Whitney U test was used to determine association between cumulative phenylephrine dose before PACU and nausea/vomiting or shivering. All analyses were conducted in SAS v. 9.3 (SAS Institute, Cary NC)

## Results

Data were collected on 40 patients between April 2012 and June 2013.

Collected data on maternal temperature, change in temperature, heart rate, mean arterial pressure, and phenylephrine dose administered are presented in Table 1. Median maternal temperature decreased with time. Although temperature did not decrease in the first 10 minutes, it steadily decreased throughout all other time points.

Univariate associations for change in maternal temperature were examined (Table 2). Maternal temperature change was associated with baseline temperature, cumulative phenylephrine dose, and time (P<0.001 for all). Subjects with a higher baseline temperature showed greater decreases in temperature. An increase of 100 mcg of phenylephrine was associated with decreased maternal temperature of 0.007°C. A 10 minute increase in time was associated a 0.04°C decrease in temperature. Additionally, MAP was not significantly associated with a change in maternal temperatures (P=0.472).

Change in maternal temperature was also examined in a multivariable model (Table 2). A greater temperature change was associated with increased baseline temperature, cumulative phenylephrine dose, time, and time by dose interaction.

**Figure 1:**
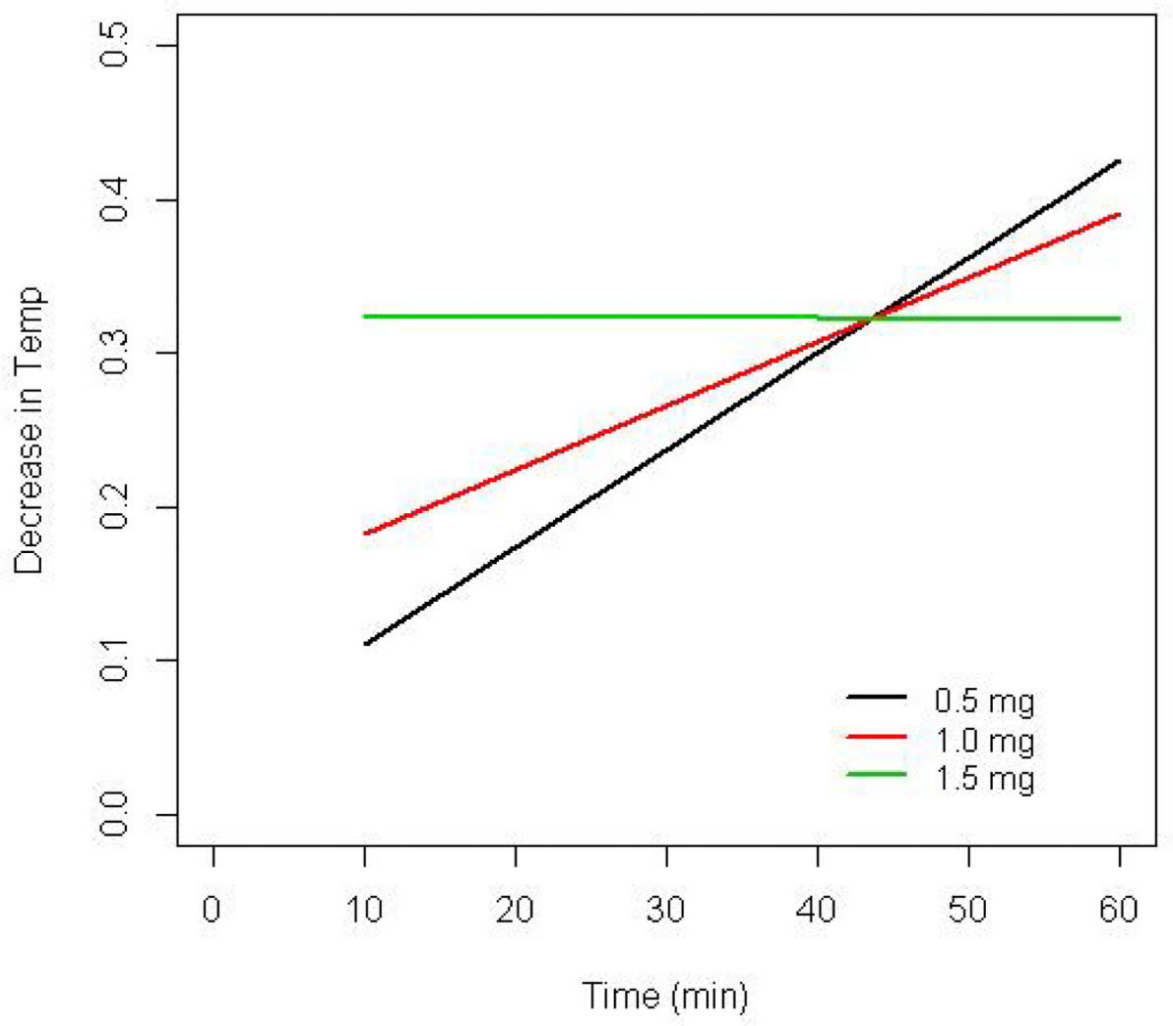
The predicted decrease in maternal temperature over time associated with 3 different phenylephrine doses based on the multivariate model presented in Table 2. Higher phenylephrine dose requirements to maintain mean arterial pressure were associated with greater decreases in temperature at earlier time points.

The predicted decrease in maternal temperature over time associated with an increasing dose of phenylephrine is shown in Figure 1. The strength of the association between cumulative phenylephrine dose and maternal temperature was strongest at earlier times. Otherwise stated, higher doses of phenylephrine at early times were observed in parturients with a greater decrease in temperature relative to lower doses; however, phenylephrine dose had less impact on temperature at later times.

Secondary endpoints are presented in Table 3. Four patients reported nausea during the course of labor and delivery. Nine patients reported shivering, six with a BSAS score of 1 and three with a BSAS score of 2. Only three of the patients reported shivering for more than 30 minutes. There were no reports of wound site infections on chart review. Estimated blood loss was 837.5 mL ± 271.2 mL and average dermatome level T4.3 ± 0.9. Cumulative phenylephrine dose was not associated with shivering during the procedure or with occurrence of nausea or vomiting (*P*=0.486 and 0.388 respectively). Receiving a higher cumulative phenylephrine dose was found to be associated with increased blood loss (*P*=0.018). A 100 μg increase in cumulative phenylephrine dose before PACU was associated with a 10 mL increase in estimated blood loss.

## Discussion and Conclusion

This investigation suggests that concomitant administration of phenylephrine during Cesarean delivery under spinal anesthesia is associated with a decreased magnitude of perioperative maternal hypothermia. Although maternal temperature did decrease, it was limited to a temperature change of no more than 0.29°C with none of the patients reaching temperatures less than 36.3°C.

Redistribution as a cause of hypothermia occurs primarily during the first hour of surgery [1]. The short surgical duration of Cesarean delivery would favor redistribution as a significant mechanism for hypothermia. A strategy to decrease redistribution hypothermia is pre-warming the patient prior to surgery to reduce the core to peripheral temperature gradient. Horn et al. [4] demonstrated that a 15 minute pre-warming prior to surgery and continuous intraoperative forced-air warming of parturients for Cesarean delivery was beneficial in maintaining maternal temperature [17]. Another strategy to decrease redistribution hypothermia that has been investigated is the administration of phenylephrine. There have been only two previous studies which have investigated the impact of phenylephrine on perioperative hypothermia. In the study by Ro et al. [13] on orthopedic surgeries performed under spinal anesthesia, the concomitant administration of phenylephrine infusion showed significant reduction in the development of core hypothermia. Ikeda et al. [18] demonstrated that use of phenylephrine as a continuous infusion decreased the magnitude of redistribution hypothermia in patients undergoing oral surgery with general anaesthesia. The thermoprotection from redistribution hypothermia was proposed to occur by vasoconstriction of the pre-capillary vasculature, mediated by α_1_ receptor activation. Our study is the first to investigate the effect of phenylephrine on maternal temperature during Cesarean delivery under spinal anesthesia and the results suggest that phenylephrine used during Cesarean delivery for maintenance of MAP may have an added beneficial effect on decreasing the magnitude of redistribution hypothermia.

The multivariable model associated the greatest decrease in maternal temperature to larger doses of phenylephrine being administered earlier in the delivery process (Figure 1). However, it is not likely that phenylephrine caused a decrease in temperature. Rather, it is plausible that patients requiring higher phenylephrine doses to maintain MAP within 20% of baseline had a greater magnitude of vasodilation due to the loss of sympathetic tonefollowing spinal placement. Increased vasodilation resulted not only in lower MAP and increased phenylephrine requirement, but also increased core-to-peripheral redistribution and consequently, heat loss.

Increasing emphasis has been placed on avoiding intraoperative hypothermia due to the significant morbidity associated with its presence. Winkler et al. [6] reported a decrease in core temperature by 0.5°C has been shown to significantly increase surgical blood loss by upwards of 200 Ml. In the current study, after controlling for baseline temperature, there was no association found between estimated blood loss and change in maternal temperature (*P*=0.644). In addition to coagulopathy, other studies have linked hypothermia to complications such as increased surgical site infections with delayed wound healing [5]. There were no reported wound infections noted upon chart review of the study patients two weeks after surgery.

Other secondary endpoints evaluated during this study included nausea, vomiting, and shivering. The incidence of nausea for subjects was 10%. Previous studies have reported rates of nausea as high as 80% and attributed this to hypotension, visceral pain and stimulation, vagal hyperactivity, use of uterotonic agents, and IV opioid administration [19]. With regard to shivering, the current study noted a 22.5% (N=9) incidence of shivering at least once during the procedure, with shivering for more than 30 minutes in 0.08% (N=3) of subjects. This is greatly reduced from reported findings of 40–60% incidence of shivering following volatile anesthetics [20], 56–60% following epidural anesthesia17, 21 and 37–40% with spinal techniques [21,22]. The paucity of these complications may be due to the thermo-protective effect of the phenylephrine infusion resulting in a marginal decrease in temperature of 0.29°C or less from baseline.

There are limitations to consider. First, there was not a control group due to the necessity to treat hypotension following spinal anesthesia. Other considerations include the lack of standardization of total IV fluids administered, IV flow rate, and absence of defined core body temperature monitoring. Additionally, we are not able to prove cause and effect with an observational study.

In conclusion, limited studies have addressed the issue of perioperative hypothermia during Cesarean delivery. Common interventions employed to prevent hypothermia during Cesarean delivery have included pre-warming the patient prior to surgery, intraoperative warmed intravenous fluids and forced air-warming; unfortunately, these interventions have not proven consistently effective [10]. This study evaluated whether phenylephrine infusion could attenuate the development of hypothermia via core-to-peripheral redistribution in parturients undergoing Cesarean delivery under spinal anesthesia. A decrease in the extent of perioperative hypothermia was observed. The attenuation of maternal hypothermia with use of continuous phenylephrine infusion is noteworthy.

## Figures and Tables

**Table 1:** Maternal temperature, change in temperature, heart rate, mean arterial pressure, and phenylephrine dose overall. Measurements presented are mean (SD).

Time (min)	Temperature °C	Δ Temperature °C	MAP	HR	Phenylephrine Dose (mcg)
Baseline	36.9 (0.23)	---	86.3 (11.2)	87.0 (15.8)	---
10	36.8 (0.20)	0.06 (0.24)	78.7 (9.76)	69.6 (11.7)	502 (180)
20	36.6 (0.19)	0.22 (0.22)	79.0 (10.9)	78.6 (14.7)	973 (397)
30	36.6 (0.18)	0.27 (0.23)	74.6 (11.0)	82.0 (18.1)	1333 (496)
40	36.6 (0.20)	0.25 (0.25)	71.9 (11.4)	83.8 (13.2)	1637 (682)
50	36.5 (0.23)	0.29 (0.23)	75.6 (11.5)	80.4 (18.7)	1961 (932)
60	36.6 (0.23)	0.20 (0.19)	73.6 (9.85)	75.4 (8.96)	2104 (1160)

min: minutes; °C: degree Centigrade; MAP: Mean Arterial Pressure in mmHg; HR: Heart Rate

**Table 2:** Impact of different variables on the change in maternal temperature in both univariate and multivariate models

	Univariate Results		Multivariate Results	
	Beta (95% CIs)	*P*	Beta (95% CIs)	*P*
Intercept			22.4 (15.7, 29.0)	<0.001
Baseline Temp (°C)	−0.60 (−0.78, −0.42)	<0.001	−0.60 (−0.78, −0.43)	<0.001
Phenylephrine Dose (μg)	−0.00007 (−0.00011,−0.000004)	<0.001	−0.00018 (−0.0028,−0.00009)	<0.001
Time (per 10 min)	−0.044 (−0.056, −0.030)	<0.001	−0.084 (−0.109, −0.060)	<0.001
Dose * Time	NA		0.042 (0.025, 0.060)	<0.001
MAP	0.0010 (−0.0017, 0.0037)	0.423	NA	

°C: degree Centigrade; μg: microgram; MAP: Mean Arterial Blood Pressure mm of Hg

**Table 3:** Patient characteristics. Dermatome level and blood loss are reported as means (± SD) and side effects are reported as n (%).

Characteristic	Value (n=40)
Dermatome level (thoracic)	T4.3 (0.9)
Blood loss (mL)	837.5 (271.2)
Nausea/vomiting (n)	4 (10.0)
Shivering (n)	9 (22.5)
